# Anxiety and depression mediate the association between chemotherapy‐induced peripheral neuropathy and fatigue: Results from the population‐based PROFILES registry

**DOI:** 10.1002/pon.5176

**Published:** 2019-07-29

**Authors:** Cynthia S. Bonhof, Lonneke V. van de Poll‐Franse, Pauline A.J. Vissers, Dareczka K. Wasowicz, Johannes A. Wegdam, Dóra Révész, Gerard Vreugdenhil, Floortje Mols

**Affiliations:** ^1^ Center of Research on Psychology in Somatic Disorders (CoRPS), Department of Medical and Clinical Psychology Tilburg University Tilburg The Netherlands; ^2^ Department of Research Netherlands Comprehensive Cancer Organisation (IKNL) Utrecht The Netherlands; ^3^ Division of Psychosocial Research and Epidemiology The Netherlands Cancer Institute Amsterdam The Netherlands; ^4^ Department of Surgery Elisabeth‐TweeSteden Hospital Tilburg The Netherlands; ^5^ Department of Surgery Elkerliek Hospital Helmond The Netherlands; ^6^ Department of Internal Medicine Máxima Medical Centre Eindhoven The Netherlands

**Keywords:** anxiety, chemotherapy‐induced peripheral neuropathy, colorectal cancer, depression, fatigue, oncology, PROFILES

## Abstract

**Objective:**

Chemotherapy‐induced sensory peripheral neuropathy (CIPN) is common among colorectal cancer (CRC) survivors. The aim of this study was to examine whether CIPN is associated with both psychological distress (ie, anxiety and depression) and fatigue and whether the relationship between CIPN and fatigue can (partly) be explained by psychological distress.

**Methods:**

All CRC survivors diagnosed between 2000 and 2009 as registered by the population‐based Netherlands Cancer Registry (Eindhoven region) were eligible for participation. Chemotherapy‐treated survivors completed questions on CIPN (EORTC QLQ‐CIPN20), psychological distress (HADS), and fatigue (FAS) on average 5.6 years after diagnosis. Simple and multiple mediation analyses were performed to examine anxiety and depression as possible mediators in the association between CIPN and fatigue.

**Results:**

Survivors with high (ie, upper 30% of scores) CIPN (n = 172) reported more anxiety and depressive symptoms and more fatigue compared with those with low CIPN (n = 299). Furthermore, among survivors with high CIPN, those who were anxious, depressed, or both reported more fatigue compared with those without psychological distress. These differences were clinically relevant. Finally, mediation analyses showed that while CIPN was directly associated with fatigue, the relationship between CIPN and fatigue was also significantly mediated by both anxiety and depression.

**Conclusions:**

CRC survivors with high CIPN report more fatigue, especially those who are also anxious and/or depressed. More research is needed on the direction of the relationship between CIPN, psychological distress, and fatigue. For now, the treatment of fatigue should also focus on addressing psychological distress, as treating fatigue alone might not be sufficient**.**

## 1 BACKGROUND

Colorectal cancer (CRC) is the third most common cancer among men and women, and the prevalence is still increasing.^1^ Due to the increasing prevalence, more patients are living with the side effects of this condition and its treatment. Chemotherapy‐induced peripheral neuropathy (CIPN), a side effect of chemotherapy, is common among CRC patients^2^ and has been found to severely impact HRQoL.^3^ Symptoms of CIPN are predominantly sensory and present as tingling, numbness, and aching or burning pain in the fingers, hands, toes, and feet.^4^ While CIPN symptoms reverse or improve in the majority of patients after chemotherapy treatment, a significant proportion of patients experience chronic CIPN.^2^ Moreover, symptoms can develop years after treatment has ended.^5^ Due to the high prevalence of CRC in combination with the lack of a well‐accepted treatment or prevention strategy against CIPN,^6^ more CRC survivors could be affected by CIPN.

Besides CIPN, fatigue is also a highly prevalent problem among cancer survivors, with prevalence rates up to 90%.^7^ Fatigue is one of the most debilitating symptom among cancer patients and has been related to a worse HRQoL,^8^, ^9^ a higher cancer recurrence,^10^ and a poorer survival.^10^ CIPN has been related to fatigue in previous research,^3^, ^5^, ^11^ but the mechanisms underlying this relationship are not well understood.

Psychological distress, that is depression and anxiety, has been found to be strongly correlated with fatigue.^7^, ^12^, ^13^ Also, while the studies on the relationship between CIPN and psychological distress are rather scarce, the majority did find that those with CIPN symptoms report more depression and anxiety.^14^, ^15^, ^16^, ^17^ Therefore, it is reasonable to argue that psychological distress could be a possible mechanism underlying the relationship between CIPN and fatigue. However, to our knowledge, this has not been examined before.

As interventions are not always successful in treating fatigue among individuals with cancer^18^ and since there is no prevention or treatment available for CIPN, knowledge on the mechanisms underlying the relationship between CIPN and fatigue is crucial for developing efficacious interventions for fatigue. Therefore, in this secondary analysis, we examined whether CIPN is associated with both psychological distress and fatigue and whether the relationship between CIPN and fatigue can (partly) be explained by psychological distress. We hypothesize that CIPN will be associated with more anxiety, depression, and fatigue and that the relationship between CIPN and fatigue will be mediated by both anxiety and depression.

## 2 METHODS

### 2.1 Settings and participants

Details of the data collection have previously been described.^3^ However, as the Netherlands Cancer Registry (NCR) is updated continuously, additional details of clinical characteristics were available for analysis in this study. In brief, this prospective, population‐based survey was set up in December 2010 by using data from the NCR, which compiles data of all individuals newly diagnosed with cancer.^19^ Everyone diagnosed with CRC between 2000 and 2009 as registered in the Eindhoven region of the NCR was eligible for participation. Those with unverifiable addresses, with cognitive impairment, who died prior to the start of the study or were terminally ill, and those with carcinoma in situ or already included in another study were excluded. One year later, the second data wave took place, which included a questionnaire on CIPN. Therefore, the data presented in this cross‐sectional study are based on the second data wave. At this time, patients were diagnosed between 2 and 12 years ago. This study was approved by the Central Committee on Research Involving Human Subjects (approval number NL23463.015.08) and the Medical Ethics Committee of the Maxima Medical Centre (approval number 0822). All survivors signed informed consent.

### 2.2 Data collection

Data collection was performed within PROFILES (www.profilesregistry.nl), which is a registry for the study of the physical and psychosocial impact of cancer and its treatment. PROFILES contains a large web‐based component and is linked directly to clinical data from the NCR. Details of the PROFILES data collection have previously been described.^20^ CRC survivors were informed of the study via a letter from their specialist if they still had follow‐up care visits in the hospital; those who did no longer have any follow‐up care received the letter from their former specialist. Nonrespondents were sent a reminder letter and paper‐and‐pencil questionnaire within 2 months.

### 2.3 Study measures

#### 2.3.1 Sociodemographic and clinical characteristics

Survivor's sociodemographic (ie, age, sex) and clinical information were available from the NCR. Comorbidity at time of the study was assessed with the adapted Self‐Administered Comorbidity Questionnaire (SCQ).^21^ Depression was excluded as a comorbidity since depression is one of the possible mediators in this study. Questions on marital status and educational level were added to the questionnaire.

#### 2.3.2 Chemotherapy‐induced peripheral neuropathy

As a previous study among the same sample of CRC survivors^3^ only found a difference in sensory peripheral neuropathy symptoms between those who received chemotherapy and those who did not, we will only focus on the sensory symptoms of CIPN in this study. Chemotherapy‐induced sensory peripheral neuropathy was assessed with the EORTC QLQ Chemotherapy‐Induced Peripheral Neuropathy^20^ (EORTC QLQ‐CIPN20).^22^ Respondents are asked how often they had experienced the specific sensory neuropathy symptom in the past week. Items are answered on a Likert scale ranging from (1) *not at all* to (4) *very much*. Scores were linearly transformed to a 0 to 100 scale, with higher scores indicating more complaints.

#### 2.3.3 Psychological distress

The Hospital Anxiety and Depression Scale (HADS) was used to assess anxiety (HADS‐A) and depressive symptoms (HADS‐D).^23^ It consists of 14 items, answered on a 4‐point Likert scale. Total scores range from 0 to 21, with higher scores indicating more anxiety or depression. A cutoff value of 8 for each subscale was used to identify a clinically relevant level of anxiety or depression.^24^ The HADS is a valid and reliable questionnaire, with mean Cronbach alpha of 0.83 (HADS‐A) and 0.82 (HADS‐D), and a sensitivity and specificity score of ≈0.80 for both subscales.^25^ In the current study, Cronbach alpha was 0.82 for both subscales.

#### 2.3.4 Fatigue

Fatigue was assessed with the Fatigue Assessment Scale (FAS),^26^ which consists of 10 items answered on a Likert scale ranging from (1) *never* to (5) *always*. Total scores range from 10 to 50, with higher scores reflecting more fatigue. The FAS has good psychometric qualities.^26^ In this study, Cronbach alpha was 0.85.

### 2.4 Statistical analyses

NCR data on sociodemographic and clinical characteristics enabled us to compare respondents, nonrespondents, and those with unverifiable addresses, using *t* tests for continuous variables and chi‐squared tests for categorical variables. The same analyses were done to compare differences in sample characteristics between chemotherapy‐treated survivors with high CIPN (ie, upper 30% of scores) and those with low CIPN (ie, the other 70% of scores). Specifically, those who were categorized into high CIPN scored greater than 11.11 on the sensory scale of the CIPN20.

Anxiety, depression, and fatigue were compared between survivors with high versus low CIPN using *t* tests for continuous outcomes and chi‐squared tests for differences in clinically relevant level of anxiety (HADS‐A ≥8) and depression (HADS‐D ≥8). In addition, among those with high CIPN, mean fatigue scores were compared between (1) survivors with high CIPN, (2) survivors with high CIPN, who are anxious, (3) survivors with high CIPN, who are depressed, and (4) survivors with high CIPN, who are both anxious and depressed. To determine whether differences could be considered clinically relevant, the minimal important differences (MIDs) for the HADS (anxiety ≥1.3, depression ≥1.4)^27^ and FAS (≥4.0) scores were used that were found in previous research.^28^ A MID is defined as the smallest difference in score of interest that patients perceive as important.^29^ To evaluate the mediating role of anxiety and depression in the relationship between CIPN and fatigue, PROCESS was used.^30^ PROCESS estimates the indirect effect and bias‐corrected confidence intervals (CIs) using bootstrapping. An indirect effect, which together with its CI indicates whether a variable mediates the relationship between variables *X* and *Y*, is considered significant when the CI does not include zero. In this study, model 4 within PROCESS was used, and all analyses were based on 5000 bootstrapping samples. Two simple mediation analyses were performed: one with anxiety as a possible mediator and one with depression as a possible mediator. A multiple mediation analysis, in which both anxiety and depression were added, was then conducted to examine whether anxiety and depression would remain significant mediators in the relationship between CIPN and fatigue, while statistically controlling for each other. In all mediation analyses, sex, age, stage, time since diagnosis, number of comorbidities, partner status, educational level, and tumor type (rectal vs colon) were included as covariables.

All tests were two‐sided and considered to be significant if *P* < 0.05. All statistical analyses were performed using SPSS 22 (IBM SPSS Statistics for Windows, Version 22.0 Armonk, NY: IBM Corps, USA).

## 3 Results

### 3.1 Sociodemographic and clinical characteristics

The response rate of this study was 83% (n = 1643). There were no differences in sociodemographic and clinical characteristics between respondents, nonrespondents, and those with nonverifiable addresses (data not shown). Of the 500 survivors who were given chemotherapy, 471 had complete data on sensory CIPN.

Of these survivors, those with high CIPN (ie, upper 30% of scores, mean sensory CIPN = 24.9) (n = 172) had significantly more comorbid conditions, more often reported having osteoarthritis, were diagnosed more recently, and were more often diagnosed with colon cancer instead of rectal cancer compared with those with low CIPN (ie, the other 70%, mean sensory CIPN = 2.8) (n = 299) (Table 1).

**Table 1:** Sociodemographic and clinical characteristics of the chemotherapy‐treated colorectal cancer survivors stratified by chemotherapy‐induced sensory peripheral neuropathy

	CRC Survivors with low CIPN (n = 299, 70%)	CRC Survivors with high CIPN (n = 172, 30%)	P Value
Age at time of survey (mean (SD))	66.4 (9.9)	66.4 (9.5)	0.99
Sex (female)	120 (40%)	77 (45%)	0.33
Partner (yes)	243 (82%)	145 (84%)	0.45
Educational level^a^			0.22
Low	33 (11%)	28 (16%)	
Middle	184 (62%)	103 (60%)	
High	81 (27%)	40 (23%)	
Number of comorbid conditions			**0.003**
0	93 (33%)	34 (21%)	
1	97 (35%)	51 (32%)	
≥2	89 (32%)	76 (47%)	
Tumor location			**0.02**
Colon	185 (62%)	125 (73%)	
Rectal	114 (38%)	47 (27%)	
Years since diagnosis (mean (SD))	6.1 (2.8)	4.7 (2.4)	**<0.001**
TNM stage			0.40
I	17 (6%)	9 (5%)	
II	48 (16%)	19 (11%)	
III	203 (68%)	125 (73%)	
IV	21 (7%)	16 (9%)	
Unknown	10 (3%)	3 (2%)	
Tumor differentiation grade			0.92
Well differentiated	26 (9%)	17 (10%)	
Moderately differentiated	185 (62%)	101 (59%)	
Poorly differentiated	40 (13%)	25 (15%)	
Unknown	48 (16%)	29 (17%)	
Comorbidities associated with PN‐like symptoms^b^			
Diabetes mellitus	41 (15%)	23 (14%)	0.91
Rheumatoid arthritis	15 (5%)	11 (7%)	0.53
Osteoarthritis	51 (18%)	51 (32%)	**0.001**

Abbreviations: SD, standard deviation; CIPN, chemotherapy‐induced peripheral neuropathy.

a Education: low (no or primary school), medium (lower general secondary education or vocational training), high (preuniversity education, high vocational training, university).

b Most frequent comorbidities associated with peripheral neuropathy.

### 3.2 Comparing high versus low CIPN on psychological distress and fatigue

CRC survivors with high CIPN reported more anxiety (*M* = 5.5, SD = 3.8 vs *M* = 4.1, SD = 3.5; *P* < 0.001) and depressive symptoms (*M* = 5.1, SD = 4.0 vs *M* = 3.9, SD = 3.4; *P* = 0.001) compared with those with low CIPN. However, these differences were not clinically relevant. Using the cutoff score of 8, the prevalence of anxiety (30% vs 19%; *P* = 0.007) and depression (27% vs 14%; *P* < 0.001) was higher among survivors with high CIPN than those with low CIPN. Regarding fatigue, those with high CIPN reported more fatigue (*M* = 23.1, SD = 7.3 vs *M* = 19.0, SD = 5.8; *P* < 0.001) than those with low CIPN. This difference was clinically relevant.

### 3.3 Anxiety and depression as mediators between CIPN and fatigue

**Figure 1:**
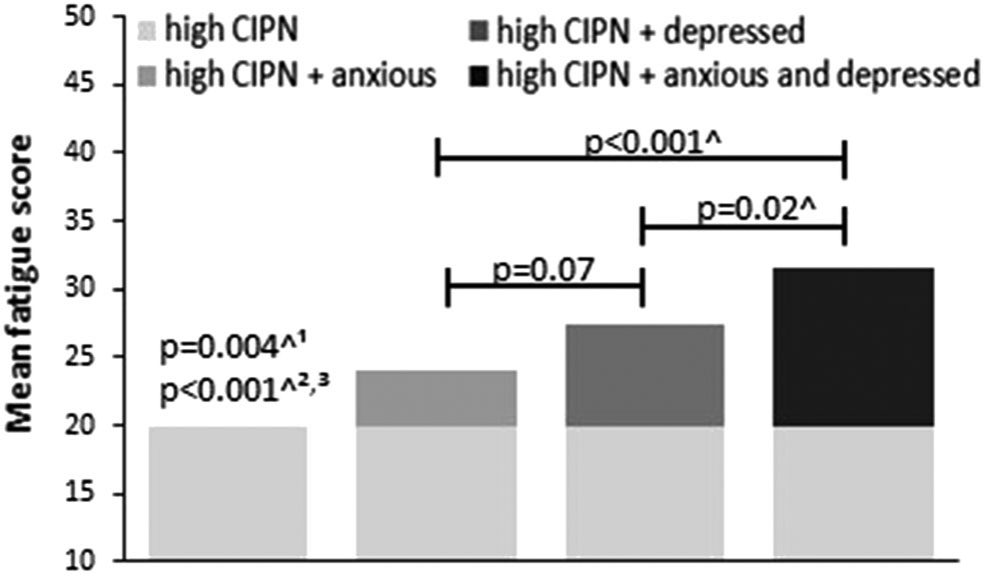
Mean fatigue (FAS) scores of colorectal cancer survivors with high chemotherapy‐induced sensory peripheral neuropathy (CIPN), stratified by anxiety and depression, or both. ^1^ High CIPN versus high CIPN + anxious. ^2^ High CIPN versus high CIPN + depressed. ^3^ High CIPN versus high CIPN + anxious and depressed. ^ Clinically relevant difference^28^

First, among those with high CIPN, fatigue scores were compared according to their anxiety and depression level (Figure 1). Compared with survivors with high CIPN only, those who were also anxious, depressed, or both reported more fatigue. Moreover, survivors who were both anxious and depressed reported more fatigue compared with those with high CIPN who were only anxious or depressed. All differences were clinically relevant.

Second, simple mediation analyses showed that a high CIPN level was directly associated with more anxiety and fatigue (Table 2). Moreover, anxiety was directly associated with fatigue as well. The bootstrap results for the indirect effect of CIPN on fatigue revealed that anxiety significantly mediated the effect of CIPN on fatigue (*B* = 1.18, 95% CI 0.54‐1.96). For depression, results showed that a high CIPN level was directly associated with more depressive symptoms and fatigue. In addition, depressive symptoms were directly related to fatigue. Finally, depression was shown to significantly mediate the effect of CIPN on fatigue (*B* = 1.08, 95% CI 0.22‐2.02).

**Table 2:** Simple mediation analyses of anxiety and depression as a mediator in the relationship between chemotherapy‐induced sensory peripheral neuropathy symptoms and fatigue (N = 424)

	Outcome Variable					
	Anxiety (M1)			Fatigue		
	B a	SE	P	B a	SE	P
CIPN^b^	1.28	0.38	<0.001	2.62	0.55	<0.001
Anxiety (M1)	‐	‐	‐	0.92	0.72	<0.001
Constant	6.20	1.93	<0.001	22.36	2.84	0.001

	Depression (M2)			Fatigue		
	*B* a	SE	*P*	*B* a	SE	*P*
CIPN^b^	0.95	0.37	0.01	2.72	0.49	<0.001
Depression (M2)	‐	‐	‐	1.14	0.07	<0.001
Constant	5.43	1.90	0.005	21.89	2.54	<0.001

*Notes*. Confounding background variables included are age, sex, time since diagnosis, number of comorbid conditions, stage, partner status, educational level, and tumor type (rectal vs colon).

Abbreviations: CIPN, chemotherapy‐induced sensory peripheral neuropathy; M, mediator.

a Values are unstandardized betas.

b High CIPN (ie, upper 30% of scores) versus low CIPN (ie, the other 70% of scores).

**Figure 2:**
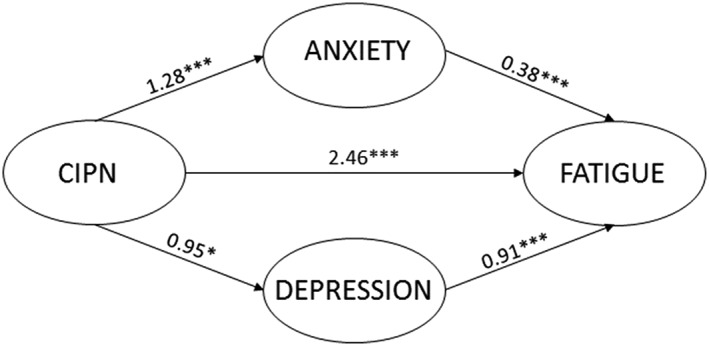
Multiple mediation analysis of anxiety and depressive symptoms as mediators in the association between chemotherapy‐induced sensory peripheral neuropathy and fatigue.Unstandardized beta's are reported. *P < 0.05, **P < 0.01, ***P < 0.001

In the multiple mediation model, in which the two possible mediators were simultaneously added to the model, both anxiety (*B* = 0.48, 95% CI 0.18‐1.01) and depression (*B* = 0.86, 95% CI 0.20‐1.69) remained significant mediators in the relationship between CIPN and fatigue (total effect = *B* = 1.34, 95% CI 0.47‐2.34) (Figure 2).

## 4 CONCLUSIONS

In this secondary analysis of a large population‐based study among long‐term chemotherapy‐treated CRC survivors, we first demonstrated that survivors with high CIPN reported more fatigue. In addition, those with high CIPN reported more anxiety and depression, which in turn increased their fatigue levels even more. Finally, both anxiety and depression were significant mediators in the relationship between CIPN and fatigue.

### 4.1 Association between CIPN, psychological distress, and fatigue

Our first aim was to examine whether CIPN was associated with both psychological distress and fatigue. Regarding psychological distress, survivors with a high CIPN level reported more anxiety and depressive symptoms compared with those with a low CIPN level. The results are in line with several prior studies, in which a positive association between CIPN and either anxiety^14^, ^15^, ^16^ or depression^14^, ^15^, ^17^ was also found**.** While we cannot determine causality due to the cross‐sectional nature of our study, previous studies have indicated that the association between CIPN and psychological distress may be bidirectional. On the one hand, CIPN symptoms could induce psychological distress due to the pain associated with CIPN and the limitations in the patient's ability to perform tasks independently.^31^ In addition, CIPN might serve as a constant reminder of having (had) cancer, which could also contribute to anxiety and depression. Reversely, people with a high level of anxiety or depressive symptoms may actually be likely to report more CIPN symptoms.^16^ One of the possible biological mechanisms that have been suggested to play a role is an increased production of proinflammatory cytokines, which has been related to both anxiety and CIPN.^32^, ^33^ Moreover, proinflammatory cytokines that are produced in an anxious condition might interfere with recovery from the nerve injury in CIPN. People with depressive symptoms may also be likely to report more CIPN, as the deficits of serotonin and norepinephrine in their brain might lead to the amplification of minor signals from the body, causing an increased attention to bodily symptoms.^34^ Also, given that antidepressants such as duloxetine have been shown to be helpful in reducing CIPN^6^ supports evidence for a biological link between psychological stress factors and CIPN. While in the current student, differences in anxiety and depressive symptoms between survivors with high versus low CIPN were all statistically significant, none were clinically relevant. This could be due to the particular sample of this study, as survivors were at least 2 years after diagnosis. Survivors could have learned to accept or at least deal with the CIPN symptoms over time. This could explain the rather small differences in anxiety and depression scores between survivors with a high and a low CIPN level. Future studies are needed that examine the association between CIPN and psychological distress shortly after, or even during chemotherapy.

Regarding the relationship between CIPN and fatigue, the results are in line with previous studies.^3^, ^5^, ^11^ Among cancer patients, CIPN has been associated with higher degrees of sleep disturbance, poor sleep quality,^14^, ^15^, ^17^ and less physical activity,^35^ which could lead to more fatigue. Elevated proinflammatory cytokines might play a role here as well, as both CIPN and fatigue have been associated with elevated proinflammatory cytokines.^33^, ^36^

### 4.2 Mediation of psychological distress in the association between CIPN and fatigue

The second aim of our study was to examine whether the relationship between CIPN and fatigue would be mediated by anxiety and depression. Both anxiety and depression were found to be associated with fatigue and to mediate the relationship between CIPN and fatigue. While prior studies did find anxiety and depression to be predictors of fatigue,^12^, ^13^ this is the first study that has examined anxiety and depression as possible mediators in the association between CIPN and fatigue. Both anxiety and depression may affect fatigue in various ways. It could be that patients with CIPN who are depressed experience more fatigue, because they either lack the motivation to exercise^37^ or because of the sleep disturbances often associated with depression.^38^ Anxious patients with CIPN may be less physically active because of the fall risk among those with neuropathy in the feet^14^ or because they may belief that too much activity could worsen CIPN symptoms. Also, the high‐stress responses that those with anxiety often experience may increase levels of fatigue. However, as existing research has yet been able to determine the direction of the relationship between psychological distress and fatigue, only tentative inferences can be made.

### 4.3 Study limitations

This study has some limitations that need to be mentioned. First, we only focused on sensory symptoms of CIPN, as a previous study in the same sample of CRC survivors only found differences in these symptoms between survivors who underwent chemotherapy and those that did not.^3^ However, the CRC survivors were 2 to 12 years after diagnosis. It is possible that CRC survivors do experience high motor and autonomic neuropathy in the first year after diagnosis and that these symptoms are associated with psychological distress and fatigue. Also, nonrespondents could have declined to participate due to CIPN symptoms in their hands or because of symptoms of fatigue or psychological distress, which could have resulted in an underestimation of the results of this study. In addition, data on the chemotherapy regimen (eg, type of chemotherapy, number of cycles) were not available, while this influences the severity of CIPN.^2^ Also, besides diabetes mellitus, rheumatoid arthritis, and osteoarthritis, we do not have information on other conditions that could have affected the neuropathy symptoms. Furthermore, while using a self‐reported questionnaire to assess CIPN is necessary due to the subjective nature of the symptoms, the lack of a clinical diagnosis of CIPN is another limitation of this study. Finally, the cross‐sectional nature of this study limits the determination of causal associations. For example, it could be that the association between psychological distress and fatigue is in fact mediated by CIPN.

Despite these limitations, this is the first study that has examined anxiety and depression as possible mediators in the relationship between CIPN and fatigue. Also, we feel that this study provides valuable new insights into the limited available data on the relationship between CIPN and anxiety, depression, and fatigue. In addition, we used the FAS to measure fatigue, which takes more elements of fatigue into account compared with the EORTC QLQ‐C30 fatigue subscale, which was mostly used in prior studies. Further, as this was a large population‐based study with a high response rate, we feel that our findings can be generalized to the general CRC survivors' population treated with chemotherapy.

### 4.4 Clinical implications

Given that this was a cross‐sectional study, no clear statements can be made regarding the causality in the relationship between CIPN, psychological distress, and fatigue. Further studies are needed that focus on the interrelationship between CIPN, psychological distress, and fatigue. For example, intervention studies aimed at improving psychological distress could be evaluated on its concomitant effect on CIPN and fatigue.

For clinical practice, this study first shows that interventions for CRC survivors with high CIPN are needed, as they report clinically relevant higher fatigue levels. Further, CRC patients with CIPN who report fatigue should be screened for psychological distress, as those with anxiety and/or depression report the most fatigue. Accordingly, in the treatment of fatigue, treatment should also focus on the presence of anxiety and depression, as treating fatigue alone might not be sufficient. Exercise and psychological interventions are recommended, as these interventions have been proven to be effective in reducing not only psychological distress and fatigue, but CIPN as well.^39^, ^40^

## FUNDING SOURCE

None.

## CONFLICT OF INTEREST

None.

## DATA AVAILABILITY STATEMENT

The data that support the findings of this study are available from the corresponding author upon reasonable request.
